# Monitoring Child Mortality through Community Health Worker Reporting of Births and Deaths in Malawi: Validation against a Household Mortality Survey

**DOI:** 10.1371/journal.pone.0088939

**Published:** 2014-02-18

**Authors:** Agbessi Amouzou, Benjamin Banda, Willie Kachaka, Olga Joos, Mercy Kanyuka, Kenneth Hill, Jennifer Bryce

**Affiliations:** 1 Institute for International Programs, Department of International Health, Johns Hopkins Bloomberg School of Public Health, Baltimore, Maryland, United States of America; 2 Malawi National Statistical Office, Zomba, Malawi; Tulane University School of Public Health and Tropical Medicine, United States of America

## Abstract

**Background:**

The rate of decline in child mortality is too slow in most African countries to achieve the Millennium Development Goal of reducing under-five mortality by two-thirds between 1990 and 2015. Effective strategies to monitor child mortality are needed where accurate vital registration data are lacking to help governments assess and report on progress in child survival. We present results from a test of a mortality monitoring approach based on recording of births and deaths by specially trained community health workers (CHWs) in Malawi.

**Methods and Findings:**

Government-employed community health workers in Malawi are responsible for maintaining a Village Health Register, in which they record births and deaths that occur in their catchment area. We expanded on this system to provide additional training, supervision and incentives. We tested the equivalence between child mortality rates obtained from data on births and deaths collected by 160 randomly-selected and trained CHWs over twenty months in two districts to those computed through a standard household mortality survey. CHW reports produced an under-five mortality rate that was 84% (95%CI: [0.71,1.00]) of the household survey mortality rate and statistically equivalent to it. However, CHW data consistently underestimated under-five mortality, with levels of under-estimation increasing over time. Under-five deaths were more likely to be missed than births. Neonatal and infant deaths were more likely to be missed than older deaths.

**Conclusion:**

This first test of the accuracy and completeness of vital events data reported by CHWs in Malawi as a strategy for monitoring child mortality shows promising results but underestimated child mortality and was not stable over the four periods assessed. Given the Malawi government's commitment to strengthen its vital registration system, we are working with the Ministry of Health to implement a revised version of the approach that provides increased support to CHWs.

## Introduction

Effective and timely tracking of progress toward the Millennium Development Goals (MDGs) is essential to address the slow gains observed in most low- and middle-income countries. The search for strategies to accelerate progress toward MDG four, which calls for a reduction in under-five mortality of two-thirds between 1990 and 2015, has highlighted the urgent need for timely measures of mortality for the assessment of the impact of child survival programs [1]–[4].

Methods for tracking under-five mortality in “real time” are needed most urgently in low-income countries where progress is slow, the burden of child mortality is high, and health systems are challenged. These countries must be able to demonstrate that the large external investments dedicated to their health system strengthening and child survival programs are producing expected declines in mortality and improvements in health conditions among children under the age of five. This requires the ability to monitor not only the inputs and processes of child survival programs, but also their outcomes and impact, for periods that are sufficiently recent and short to allow timely feedback to improve these programs.

A further challenge is that these monitoring systems must be established in contexts where vital registration systems for tracking vital events are weak or non-existent, and health management information systems are not yet able to produce the data needed to assess impact [5]–[8]. Demographic surveillance systems can produce high-quality mortality data, but usually focus on relatively small populations that become increasingly non-representative of the national population over time. National surveys, such as Demographic and Health Surveys (DHS) and Multiple Indicator Cluster Surveys (MICS), are the most widely used sources of data on child mortality, but produce mortality estimates that are generally averages over the five years before the survey for national level estimates, and over the ten years before the survey for sub-national level estimates. These surveys are inadequate to assess recent changes [9]. Innovative approaches for monitoring changes in child mortality in “real-time” are therefore needed urgently.

The United Nations Commission on Accountability for Women's and Children's Health underscored the critical need for countries to develop or strengthen systems for tracking vital events, to provide a platform for assessing levels and trends in maternal, neonatal and child mortality on a short term basis[10]. The challenge faced by low-income countries and the international scientific and public health communities is to develop, validate and implement new approaches for monitoring child mortality in settings where health systems are challenged by human, financial and logistical resource shortages and vital registration systems are not yet established or functioning at optimal level. Few studies have implemented and tested the accuracy of approaches for tracking vital events in communities as a strategy for monitoring mortality, and these studies were limited to very small communities [11].

With support from the Government of Canada, the Institute for International Programs (IIP) at Johns Hopkins University is implementing a “real-time mortality monitoring” (RMM) project in five countries in Africa (Ethiopia, Ghana, Malawi, Mali and Niger), working closely with in-country research partners. The RMM project aims to develop and test low cost and sustainable methods for measuring mortality among children under the age of five in settings without fully functional vital registration systems.

We began the project with a consultative process to identify feasible innovative approaches, involving international experts in mortality measurement and the tracking of vital events at community level [12]. We then consulted with country stakeholders, including Ministries of Health and other partners, and assessed existing opportunities to develop and test these innovative approaches. In each country, we identified at least two promising approaches and implemented each for a period of at least twelve months. The resulting mortality data were then compared to results produced by a rigorous household survey or census to determine the accuracy of the approach. Approaches that produce mortality measures that fall within 20% of the gold-standard measures are judged acceptable.

In Malawi, we worked with the National Statistics Office (NSO) to identify locally appropriate and promising approaches. We implemented each approach for two years (2010–2011) in two of Malawi's 28 districts, Balaka and Salima.

One of the RMM approaches implemented in Malawi is based on having community health workers (CHWs) in a random sample of catchment areas in each district record pregnancies, births and deaths in the government village health registers (VHRs). The CHWs, referred to locally as Health Surveillance Assistants (HSAs), received special training and incentives to record these events in their VHRs. The approach was implemented from January 2010 through December 2011, and a mortality survey was conducted between October 2011 and February 2012 to provide validation measurements of child mortality for the same periods. In this paper, we describe how the RMM approach was implemented and assess the validity of the mortality estimates obtained in comparison to estimates from the household mortality survey.

## Methods

### Ethics Statement

Ethical clearance for the project was obtained in the United States from the Johns Hopkins Bloomberg School of Public Health (JHSPH)'s Institutional Review Board, and in Malawi from the National Health Sciences Research Committee. For the household survey, oral informed consent was obtained from each participant. The consent forms were translated into the local language, "Chichewa". The IRB at JHSPH waived the need for written consent from the study participants given the low literacy of the population under study. Approval letters are available upon request.

### Setting

Malawi is a poor country in southern Africa with a population of 13.1 million according to the 2008 population census[13]. It is among countries with the poorest health indicators in the region. The 2010 Demographic and Health Survey reported an under-five mortality rate of 112, a maternal mortality ratio of 675 per 100,000 live births, and high fertility with an average number of children per woman of 5.7[14]. Malawi has one of the highest rates of HIV/AIDS prevalence in Africa, estimated in 2010 to be 10.6% among adults. Despite this high burden, Malawi has made good progress in child survival in recent years, and is one of few countries in sub-Saharan Africa reported to be on track to achieving MDG 4, with an annual rate of reduction in under-five mortality of 5.6%[1], [3].

The country has made a commitment to improving health indicators through the adoption of policies enabling implementation of high-impact interventions in maternal, newborn and child health and HIV/AIDS. In 2004, Malawi adopted a Health Sector Wide Approach (SWAp), with a five-year joint Program of Work (2004–2009) for delivery of an Essential Health Package supported by multiple donors. This resulted in the development of a Road Map for Accelerating Attainment of the MDGs on Maternal and Newborn Health in 2005 and the adoption of the Integrated Management of Childhood Illness Strategy (IMCI) for Accelerated Child Survival and Development (ACSD) in 2006.

In addition to the IMCI strategy, Malawi has also adopted an integrated community case management (CCM) strategy to increase access to correct treatment for pneumonia, malaria and diarrhea. A key element of this strategy is the Health Surveillance Assistant (HSA), a level of personnel with about 10 years of education and 10 weeks of basic health training. HSAs are allocated to defined geographic areas of approximately 1,000 population each. CCM is delivered by CCM-trained HSAs who also conduct preventive care and community monitoring[15]. With support from the Global Fund, in 2008 the country doubled the number of HSAs from about 5,500 to 11,000, with the goal of reaching a ratio of one CHW per 1,000 population nationally[15].

The expansion of the HSA cadre provided a unique opportunity to test an RMM approach relying on these community health workers for reporting of pregnancies, births and deaths for monitoring of maternal and child mortality. Almost all districts in the country are covered by HSAs, and if this cadre is maintained in a sustainable way, it could represent an important asset for the development of a viable vital registration system in the country.

We tested the approach in the districts of Balaka and Salima. District selection criteria included high under-five mortality, high fertility, easy access for the study team, full coverage of HSAs deployed, and average population size based on the distribution of district population size across the country. Balaka is located in the southern region of the country, with a population of 316,748 according to the 2008 population census. Salima is in the central region, with a population of 340,327. According to the 2010 DHS, Balaka and Salima have high under-five mortality at 125 and 150 deaths per 1000 live births, and high fertility at 6.0 and 6.6 children per woman, respectively[14]. In 2010, a total of 280 HSAs were deployed and working in Balaka, and 344 in Salima.

### HSAs and Implementation of the RMM Project

HSAs are recruited after completion of 10 years of education (junior certificate level). They receive basic health service training of ten weeks' duration, and are then assigned to a specific catchment area in a district[15]–[17]. HSAs are not necessarily drawn from among community members, although they are required to reside in their catchment area after deployment. About 60% of HSAs in 2008 were reported by the Ministry of Health to be male. HSAs report to the nearest health center and are supervised by Environmental Health Officers (EHO), Community Health Nurses (CHN) and Senior HSAs (HSAs who have spent at least two years at their post).

HSAs monitor an average population of 1000 people, and are responsible for completing government-provided Village Health Registers (VHRs). They are supposed to complete the VHR by first conducting a complete enumeration of their catchment area population, and then routinely recording pregnancies, births, deaths, and other information including antenatal care visits, children's immunization status, growth monitoring, and the status of water and sanitation facilities. The HSAs are supposed to develop a weekly work plan that includes household visits, inspection of public facilities, and the conduct of outreach clinics and village feedback meetings. The RMM project strengthened the performance of the monitoring tasks by providing supplemental training to the HSAs, along with incentives and increased supervision.

We started the RMM work by conducting a formative research study using qualitative methods in June, 2009 in the districts of Kasungu and Mangochi. The study had two objectives: 1) to provide the information needed to develop clear and effective procedures for HSAs' recording of vital events, including initial training, supervision and support; and 2) to define possible alternative strategies for collecting data on vital events in the local context. The formative research allowed us to learn more about HSAs, their duties in terms of identification and reporting of pregnancies, births and deaths, the reporting barriers and challenges, supervision issues and other alternative ways to identify vital events within the community. The formative research showed clearly that use of the VHRs was an appropriate community approach to RMM, and one that was likely to be sustainable and cost-effective in the Malawi context. These conclusions were confirmed in a meeting of Ministry of Health and other stakeholders in October 2009.

We then organized and conducted one-day sensitization meetings in Balaka and Salima before the start of actual implementation. Participants included District Health Officers (DHOs), District Environmental Health Officers (DEHO), Traditional Authorities (TA), Members of the District Assembly (DA), representatives of the national MOH and representatives of local NGOs. The objectives of the sensitization meetings were to inform district officials and leaders about the plans for implementation of an activity related to the monitoring of mortality among children under the age of five, and to generate support for the project among district officials and stakeholders. The project received support and encouragement from the district officials and participants at the sensitization meetings.

Using an equivalence test[18], we estimated that a sample of at least 80 HSA catchment areas, in each of the two selected RMM districts, was required to reject the hypothesis of non-equivalence between the mortality rate computed from the HSAs' data and the rate generated from the validation survey with 24,000 households, using 80% power and a margin of error of 20% of the survey mortality rate. To select the catchment areas, we conducted a complete mapping of all HSA catchment areas in each of the two districts, and developed a sampling frame from which 80 catchment areas were randomly selected using a simple random sampling procedure. HSAs assigned to these catchment areas and their supervisors were then identified, trained and supported to complete their VHRs correctly and to extract data on pregnancies, births and deaths from the VHRs every month onto simple summary forms provided to them by the RMM project staff. The training required one day in each district and was led by the regular district HSA trainers. Following the training and before the start of data collection, each HSA was provided with at least one new VHR, a backpack, and a cell phone and airtime.

In each district, about 15 HSA supervisors were identified. These individuals were the regular government-mandated HSA supervisors who serve as Environmental Health Officers (EHO) or Assistant Environmental Health Officers under the overall supervision of the District Environmental Health Officer (DEHO). The HSA supervisors also participated in a one-day training on supervision procedures, data recording and extraction from VHRs, and data flow processes. Additionally, each supervisor was required to conduct one-on-one refresher training of each HSA under his/her supervision. A district coordinator was appointed to manage RMM activities. RMM HSAs and their supervisors received quarterly allowances of cellphone airtime. A transportation allowance was provided to each supervisor to facilitate the supervision visits. Data extraction from VHRs started in January 2010 and has continued to date; in this analysis we focus on the vital events recorded through September 2011 before the mortality survey was carried out.

### Data Collection and Supervision

As indicated above, HSAs are expected to identify pregnancies, births, and deaths within their catchment areas and to record the information in their VHR as a part of their routine duties. The performance of HSAs participating in the RMM project was monitored and reinforced via increased training, supervision, quarterly review meetings and incentives. The HSAs extract the information from the registers every month using a standard RMM extraction form (Web Annex S1). To familiarize themselves with their community, HSAs began their RMM activities by listing members of each household within their catchment area in the VHR. Each household and family member was assigned a code, represented by a sequence of 11 digits with the zone (1 digit), the district (1 digit), the traditional authority (2 digits), the Group Village Head (2 digits), the household (3 digits), and the household member (2 digits). HSAs provided the completed extraction form to the responsible supervisor, who checked the data and in turn provided the form to the district RMM coordinator. A photocopy was made and sent to NSO. NSO collected the RMM extraction forms every month from the district and maintained a spreadsheet to monitor HSA reporting. A dedicated data editor at NSO checked each form, edited it, and followed up on any errors either by calling the HSA or sending a copy of the form back for correction. Data entry was completed soon after editing using CSPro[19]. Double independent data-entry was performed to minimize errors, and any differences were reconciled through review of the original data. Data were then transferred into STATA 12.0 for analysis[20].

The district RMM coordinator assigned each HSA participating in RMM to a specific supervisor at the health center. The supervisor checked the HSA's performance in completing the extraction forms and recording the births and deaths in the VHR, and made corrections as needed. The supervisor also provided immediate feedback to the HSA and conducted on-the-spot retraining in RMM procedures. Data review meetings were held regularly during the study period in both districts, with participation by the RMM HSAs, Ministry of Health officials, the District Health Officer, the HMIS officer, and other partners such UNICEF and WHO. During the review meeting, reports were made on progress in HSA reporting of pregnancies, births and deaths, and feedback was provided. HSAs and supervisors were given the opportunity to discuss challenges, issues, and to suggest solutions. NSO used the data review meetings as an opportunity to provide refresher training on RMM procedures and the data flow process, and to reinforce the importance of ensuring data quality in terms of accuracy, reliability, and completeness.

### Validation Data

Ideally, gold-standard measures of child mortality would rely on vital registration data that provide real-time information on dates of births and deaths. However, accurate and complete vital registration data are not available in Malawi, and it was not feasible to establish such for the purposes of this study. We therefore used direct mortality measurement through a high-quality household survey as our reference standard. The survey collects complete birth history data from women aged (15–49), including the date of birth of each child ever born alive, and the date or age at death for children deceased. In absence of accurate vital statistics, household surveys with full birth histories are the most internationally accepted and widely used approach for collecting required data for direct mortality measurement.

We conducted a mortality survey in Balaka and Salima districts from October 2011 through February 2012 to validate the RMM approach. The survey was carried out in 12,000 households in each district, reflecting the sample size required to reject the hypothesis of non-equivalence between the mortality rates derived from the RMM approach and the mortality survey, with 80% power. RMM mortality rates falling within 20% of the validation mortality rates were assumed to be acceptable.

The survey used a stratified two-stage cluster sampling procedure, with stratification by district and clusters based on 2008 population census enumeration areas (EAs). In the 2008 census frame, there were 293 EAs in Balaka and 435 EAs in Salima. Given a sample size of 12,000 household in each district, a total of 343 clusters was needed in each district, with 35 randomly-selected households in each cluster. The first stage involved a systematic random sampling of clusters of households, the clusters being EAs in Salima, but EAs or subdivided EAs in Balaka. In Balaka, the total number of EAs in the district was lower than the total required, so we subdivided large EAs, and all clusters were sampled. A full listing of all households in each selected EA was completed to generate an updated sampling frame of households. At the second stage, thirty five households were randomly selected to participate in the interview.

A short household questionnaire was used to interview the head of each sampled household to obtain the list of all members of the household and their demographic characteristics such as age and sex. This list allowed identification of women 15–49 eligible for interview. Full birth histories were collected from all women aged 15–49 in each selected household. The quality of the data was assessed thoroughly before the mortality analysis was conducted. Web Annex S2 provides full details on the survey procedures and the results of the data quality assessment, which indicated that the data were of good quality and could support the analysis.

Careful training and field supervision of interviewers were used to prevent the types of measurement errors known to occur during the collection of full birth history data. These errors include potential recall bias, errors in age and date declaration and omission of births and deaths. The period of mortality assessment for the current study covers the eighteen months before the survey, and is therefore recent enough to minimize recall errors in event reporting.

### Analysis

Data on births and under-five deaths reported by HSAs in the period from January 2010 to September 2011 were included in the analysis. We calculated the number of births and neonatal, infant and under-five deaths for rolling periods of twelve months beginning in January, April, July and September 2010. The number of births and deaths were adjusted for missing reports in specific months by imputing the average number of such events (births or deaths) reported by HSAs across months.

We computed neonatal, infant and under-five mortality rates by dividing the number of such deaths reported in a specific period by the total number of births for the same period. These measures correspond to conventional calculations of neonatal and infant mortality, but tend to under-estimate under-five mortality slightly compared to an approach based on life table procedures.

The validation analysis was conducted in two steps. We first estimated the level of completeness of the births and deaths reported by HSAs. We calculated the expected numbers of births and under-five deaths for each annual period defined above. Using the full birth history data from the validation survey, we computed crude birth rates for each annual period. Each rate was multiplied by the total population of the RMM HSAs' catchment areas to estimate the total number of births expected to be reported by HSAs. The expected number of under-five deaths was then estimated by multiplying the expected number of births by the under-five mortality rate computed from the validation survey. The completeness of reporting of births and deaths by HSAs was then calculated as the ratio of the total number of births and under-five deaths reported by HSAs to the expected number based on the validation survey.

In the second step, we compared the estimates of mortality rates from the HSA data directly to the rates obtained from the mortality survey. To ensure comparability, mortality rates from the mortality survey were computed using the same procedure used for the HSA data. We computed standard errors of the mortality rates from both datasets using the Jackknife repeated replications procedure[21]. The equivalence of the mortality rate from the HSA data and that of the mortality survey was assessed by computing the ratio of both rates and its standard error using the Delta method[18]. We then computed the 95% confidence interval of the mortality ratio. Based on a tolerance margin of 20% of the survey mortality rate, we rejected the hypothesis of equivalence between the two mortality rates if the upper bound of the 95% confidence interval of the ratio was less than 0.80 (indicating significant under-estimation) or the lower bound was greater than 1.20 (indicating significant overestimation)[18]. All analyses drawing on the mortality survey data were adjusted for sampling weights to take into account the sampling design of the survey. The analysis did not account for possible migration effects on the HSA reporting of the events or any recall error in the household survey.

## Results

**Figure 1:**
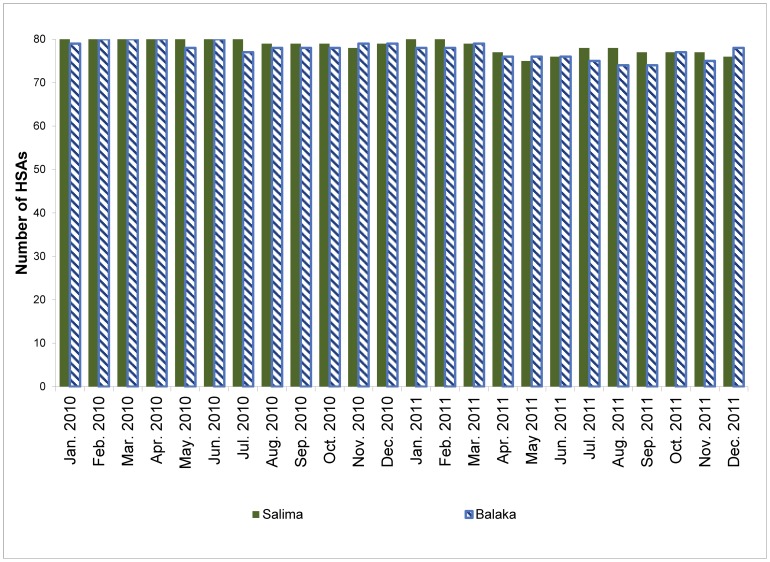
Number of HSAs who reported data by month from January 2010 to December 2011 by district (number of HSAs  = 80 per district).

Figure 1 shows the number of HSAs who reported data on vital events by month, from January 2010 to December 2011. The level of reporting was very high over this period, with over 93% of HSAs in Balaka and 95% in Salima reporting data every month. The level of reporting was almost 100% in the first year of implementation, but dropped slightly in the second year due to turnover among HSAs. Although not shown in the figure, timely reporting also declined over the study period. This was due in part to HSA turnover, but also to decreases in the level of supervision at district level and delays in the transfer of data forms, both from HSAs to their supervisors and from supervisors to the district RMM manager.

Table 1 presents the total number of births and neonatal, infant and under-five deaths reported by HSAs for sequential twelve-month periods starting each quarter from January 2010 to January 2011. The monthly number of births and under-five deaths reported are included in Web Annex S3. Except for the first twelve month period from January 2010 to December 2010, the number of births and deaths reported declined steadily over the next four periods in both districts. For both districts combined, the sex ratios at birth are all below 100 males per 100 females. Given that sex ratios in human populations are generally between 102 and 107 males per 100 females, these ratios might be taken as indicating relative omission of male births relative to female births. However, the results are consistent with the ratios observed both in the validation survey and in the 2010 Malawi Demographic and Health Survey.

**Table 1:** Number of births and childhood deaths reported by HSAs during twelve-month periods beginning between January 2010 and January 2011, by district, sex ratios at birth and ratios of neonatal and infant deaths to under-five deaths from HSAs' data and the mortality survey.

Annual periods	Number of births reported by HSAs (unadjusted for missing reports)					Sex ratio at birth (%)*				Number of deaths reported by HSAs						Ratio Neonatal to under-five deaths (%)				Ratio infant to under-five deaths (%)								
	Total	Males	Females	Missing		HSAs data		Validation survey		Neo-natal	Infant			Under-five		HSAs data		Validation survey		HSAs data			Validation survey					
										**BALAKA**																		
Jan 2010–Dec 2010	1926	909	1013	4		89.7		99.3	48				81		162	29.6		32.2		50.0			65.7					
Apr 2010–Mar 2010	1985	934	1050	1		89.0		92.3	55				88		168	32.7		38.3		52.4			68.8					
Jul 2010–Jun 2010	1913	906	1006	1		90.1		92.3	48				77		135	35.6		44.7		57.0			72.9					
Oct 2010–Sep 2011	1751	816	935	0		87.3		91.8	32				58		100	32.0		46.5		58.0			76.7					
Jan 2011–Dec 2011	1573	739	830	4		89.0		NA	36				55		86	41.9		NA		64.0			NA					
										**SALIMA**																		
Jan 2010–Dec 2010	2338	1175	1152	11		102.0		103.0	60				128	209		28.7		39.5		61.2			64.3					
Apr 2010–Mar 2010	2398	1185	1208	5		98.1		100.0	62				122	200		31.0		37.8		61.0			62.5					
Jul 2010–Jun 2010	2355	1185	1164	6		101.8		100.0	54				98	167		32.3		34.1		58.7			65.8					
Oct 2010–Sep 2011	2313	1167	1137	9		102.6		97.2	34				65	100		34.0		33.4		65.0			64.4					
Jan 2011–Dec 2011	2231	1098	1124	9		97.7		NA	30				58	90		33.3		NA		64.4			NA					
									**TOTAL**																			
Jan 2010–Dec 2010	4264	2084	2165	15		96.3		101.0	108				209	371		29.1		35.8		56.3			65.0					
Apr 2010–Mar 2010	4383	2119	2258	6		93.8		95.8	117				210	368		31.8		38.0		57.1			65.5					
Jul 2010–Jun 2010	4268	2091	2170	7		96.4		95.8	102				175	302		33.8		39.5		57.9			69.5					
Oct 2010–Sep 2011	4064	1983	2072	9		95.7		94.4	66				123	200		33.0		40.3		61.5			70.9					
Jan 2011–Dec 2011	3804	1837	1954	13		94.0		NA	66				113	176		37.5		NA		64.2			NA					

*Number of males per 100 females births.

Table 1 also presents the ratio of neonatal to under-five deaths and the ratio of infant to under-five deaths separately for data from the HSAs and from the validation survey. These ratios allow an assessment of the age patterns of deaths and of possible under-reporting of deaths within age groups. In both districts, the proportion of neonatal deaths out of all under-five deaths reported by HSAs was consistently lower than that observed in the validation survey. For example, during the year 2010, 29% of under-five deaths reported by HSAs occurred in the neonatal period (respectively 30% and 29% in Balaka and Salima), compared to 36% in the validation survey (respectively 32% and 40% in Balaka and Salima). This suggests under-reporting of neonatal deaths relative to deaths between 1 and 59 months in both districts. Similar results are observed for the proportion of infant deaths among all under-five deaths, with a lower proportion reported by HSAs than found in the validation survey. Thus, HSAs appear more likely to identify older deaths than younger deaths, with higher under-reporting of neonatal deaths. There were no systematic differences in the reporting of deaths by sex of the child.

In Table 2, we compare the total number of births and under-five deaths reported by the HSAs to the expected number of births and under-five deaths computed based on the validation survey and the estimated total population of the HSAs' catchment areas. Columns (7) and (10) present the ratio of reported to expected events. Overall, there appears to be serious under-reporting of births (over 40%). For both districts combined, HSAs reported only 50% to 57% of the births expected on the basis of the household survey. Under-reporting of births was more severe in Balaka district, where HSAs reported between 42% and 50% of expected births, than in Salima district, where HSAs reported between 59% and 67% of expected births. In both districts, reporting of births declined slightly over the four study periods.

**Table 2:** Comparison of births and under-five deaths reported by HSAs to expected births and deaths based on validation survey.

Period	Total estimated population of RMM areas	Estimated Crude birth rate (Validation Survey, per 1000)	U5MR (validation Survey, per 1000)		Births			Under-five deaths		
					Expected number of births	Number of births reported by HSAs	Ratio births reported by HSAs to expected births (%)	Expected number of U5 deaths	Number of U5 deaths reported by HSAs	Ratio U5 reported by HSAs to expected U5 deaths (%)
	**BALAKA**									
Jan 2010–Dec 2010	108317	36.7		99.2	3970	1952	49.2	394	164	41.6
Apr 2010–Mar 2011	108317	37.5		92.2	4057	2019	49.8	374	170	45.5
Jul 2010–Jun 2011	108317	39.8		92.0	4311	1966	45.6	396	139	35.0
Oct 2010–Sep 2011	108317	40.3		90.4	4364	1822	41.8	394	105	26.6
	**SALIMA**									
Jan 2010–Dec 2010	95424	37.0	107.9		3532	2353	66.6	381	210	55.1
Apr 2010–Mar 2011	95424	38.6	108.1		3688	2415	65.5	399	201	50.4
Jul 2010–Jun 2011	95424	39.8	99.4		3795	2401	63.3	377	170	45.1
Oct 2010–Sep 2011	95424	41.8	86.7		3987	2371	59.5	346	104	30.0
	**TOTAL**									
Jan 2010–Dec 2010	203741	36.8	103.3		7502	4305	57.4	775	374	48.2
Apr 2010–Mar 2011	203741	38.0	99.8		7744	4434	57.3	773	371	48.1
Jul 2010–Jun 2011	203741	39.8	95.4		8106	4367	53.9	773	309	39.9
Oct 2010–Sep 2011	203741	41.0	88.6		8351	4194	50.2	740	209	28.2

HSAs also severely under-reported under-five deaths relative to the household survey, and deaths were more likely to be missed than births. In both districts, HSAs reported less than 50% of the under-five deaths expected on the basis of the survey (43% to 25%). Similar to births, a lower proportion of under-five deaths were reported in Balaka (41% to 25%) than in Salima (48% to 25%). In addition, reporting of under-five deaths declined much faster over the four study periods than reporting of births. This excess under-reporting of under-five deaths relative to births means that the resulting mortality rates will also be under-estimated.

Tables 3, 4 and 5 present rates and ratios for under-five, infant and neonatal mortality respectively, with corresponding 95% confidence intervals, estimated using HSA records and the validation survey. The under-five mortality rate generated by the HSA data consistently under-estimated the level expected on the basis of the household survey for both districts combined and separately for each district. For both districts combined, the level of under-estimation varies from 16% to 44%. However, the statistical equivalence test based on the 95% confidence intervals of the ratio of the under-five mortality rate from HSA data to that of the survey suggests that the equivalence of the rates cannot be rejected for the first two periods (January 2010-December 2010 and April 2010-March 2011), where the ratio was 0.84 (95%CI: [0.71,1.00] and [95%CI: 0.70,1.00]) and the third period (July 2010-June 2011) where the ratio was 0.74 (95%CI:[0.62,0.89]). However, the under-five mortality rates generated from the HSA data declined quickly from the first period to the last, reaching only 56% (95%CI: [0.46,0.68]) of the survey mortality rate for the last period, a rate that is statistically non-equivalent to that produced by the survey. This rapid decline arose from a faster drop in the reports of deaths relative to births.

**Table 3:** Under-five mortality rates from HSA records and validation survey, the ratio of the two rates and corresponding 95% confidence intervals.

Annual period	Under-five mortality rate (per 1000)					
	HSAs data		Validation survey		Ratio of mortality rate, HSAs data to validation Survey	
	Rate	95%CI	Rate	95%CI	%	95%CI
	BALAKA					
Jan 2010–Dec 10	84.2	(67.4,101.0)	99.2	(82.8,115.6)	84.9	(65.7,109.6)
Apr 2010–Mar 11	84.6	(66.0,103.3)	92.2	(76.5,107.9)	91.8	(69.7,120.8)
Jul 2010–June 11	70.8	(55.5,86.1)	92.0	(76.2,107.7)	77.0	(58.6,101.0)
Oct 2010–Sep 11	57.7	(45.4,70.1)	90.4	(73.8,106.9)	63.9	(48.4,84.4)
	SALIMA					
Jan 2010–Dec 10	89.3	(74.3,104.4)	107.9	(92.5,123.2)	82.8	(66.6,103.0)
Apr 2010–Mar 11	83.3	(68.7,97.9)	108.1	(93.1,123.1)	77.0	(61.8,96.1)
Jul 2010–June 11	70.8	(57.2,84.4)	99.4	(84.1,114.6)	71.3	(55.9,90.8)
Oct 2010–Sep 11	43.7	(33.6,53.8)	86.7	(73.2,100.2)	50.4	(38.3,66.3)
	TOTAL					
Jan 2010–Dec 10	87.0	(75.9,98.1)	103.3	(91.4,115.1)	84.2	(71.1,99.8)
Apr 2010–Mar 11	83.9	(72.4,95.5)	99.8	(88.2,111.3)	84.1	(70.4,100.5)
Jul 2010–June 11	70.8	(60.7,80.9)	95.4	(83.9,107.0)	74.2	(61.7,89.3)
Oct 2010–Sep 11	49.8	(41.9,57.7)	88.6	(78.2,99.1)	56.2	(46.3,68.3)

**Table 4:** Infant mortality rates from HSA records and validation survey, the ratio of the two rates and corresponding 95% confidence intervals.

Annual period	Infant mortality rate (per 1000)							
	HSAs data		Validation survey		Ratio of mortality rates, HSAs data to validation Survey			
	Rate	95%CI	Rate	95%CI	Ratio			95%CI
	BALAKA							
Jan 2010–Dec 10	42.1	(29.4,54.8)	68.0	(54.1,81.9)			62.0	(43.3,88.8)
Apr 2010–Mar 11	44.4	(30.6,58.1)	64.5	(50.1,78.9)			68.8	(47.2,100.2)
Jul 2010–June 11	40.3	(28.7,51.9)	64.3	(50.4,78.2)			62.7	(44.0,89.5)
Oct 2010–Sep 11	33.4	(23.5,43.3)	64.0	(50.0,78.0)			52.2	(36.3,75.2)
	SALIMA							
Jan 2010–Dec 10	54.7	(44.7,64.7)	71.8	(58.0,85.6)			76.2	(58.6,99.2)
Apr 2010–Mar 11	50.8	(40.6,61.1)	70.2	(56.7,83.8)			72.4	(54.9,95.4)
Jul 2010–June 11	41.6	(32.0,51.2)	63.1	(50.1,76.1)			65.9	(48.5,89.5)
Oct 2010–Sep 11	28.4	(20.8,35.9)	53.9	(42.4,65.4)			52.7	(37.6,73.7)
	TOTAL							
Jan 2010–Dec 10	49.0	(41.1,57.0)	69.8	(60.0,79.6)			70.2	(56.8,86.9)
Apr 2010–Mar 11	47.9	(39.5,56.2)	67.2	(57.1,77.4)			71.2	(56.7,89.5)
Jul 2010–June 11	41.0	(33.6,48.4)	63.7	(54.1,73.4)			64.4	(51.0,81.2)
Oct 2010–Sep 11	30.6	(24.5,36.6)	59.2	(50.5,67.9)			51.6	(40.5,65.9)

**Table 5:** Neonatal mortality rate from HSA records and validation survey, the ratio of the two rates and corresponding 95% confidence intervals.

Annual period	Neonatal mortality rate (per 1000)						
	HSAs data		Validation survey		Ratio of mortality rates, HSAs data to validation Survey		
	Rate	95%CI	Rate	95%CI	%		95%CI
	BALAKA						
Jan 2010–Dec 10	25.0	(14.8, 35.1)	33.4	(24.1, 42.6)	74.92		(46.1, 121.7)
Apr 2010–Mar 11	27.7	(17.0, 38.5)	35.9	(24.7, 47.1)	77.21		(47.3, 126.1)
Jul 2010–June 11	25.1	(15.7, 34.5)	40.1	(28.4, 51.8)	62.71		(39.3, 100.2)
Oct 2010–Sep 11	18.5	(11.1, 25.9)	39.4	(27.7, 51.1)	47.03		(28.8, 76.9)
	SALIMA						
Jan 2010–Dec 10	25.6	(18.6, 32.7)	44.8	(33.7, 55.9)	57.28		(39.7, 82.6)
Apr 2010–Mar 11	25.8	(18.5, 33.2)	43.1	(32.6, 53.6)	59.93		(41.4, 86.8)
Jul 2010–June 11	22.9	(16.1, 29.7)	33.1	(23.8, 42.4)	69.16		(46.1, 103.6)
Oct 2010–Sep 11	14.8	(8.7, 20.9)	28.0	(20.1, 35.8)	52.94		(32.3, 86.7)
	TOTAL						
Jan 2010–Dec 10	25.3	(19.4, 31.3)	38.7	(31.5, 45.9)		65.46	(48.7, 88.0)
Apr 2010–Mar 11	26.7	(20.4, 33.0)	39.3	(31.2, 47.4)		67.87	(49.8, 92.5)
Jul 2010–June 11	23.9	(18.3, 29.5)	36.8	(28.9, 44.7)		64.91	(47.4, 88.9)
Oct 2010–Sep 11	16.4	(11.7, 21.1)	34.0	(27.1, 40.8)		48.36	(34.2, 68.4)

Similar results are observed for infant and neonatal mortality rates, shown in tables 4 and 5. Consistent with the earlier results regarding numbers of deaths, the under-estimation of neonatal mortality rates is highest relative to the survey data, followed by that of infant mortality rates, varying from 35% to 52% for neonatal mortality and from 30% to 48% for infant mortality. For infant mortality, the under-estimation was greater in Balaka than in Salima, whereas the reverse is observed for neonatal mortality.

## Discussion

Growing attention to accountability and progress monitoring for programs addressing women's and children's health has thrown into relief the need for real-time data on under-five mortality. This need cannot be met without innovative, simple, low-cost approaches that build on existing country systems and structures to produce data that program implementers can use to assess the extent to which programs are producing their expected impact. As part of the RMM project supported by Canada, we tested one such approach in Malawi, building on an existing cadre of community health workers, referred to as Health Surveillance Assistants or HSAs. We trained and supported randomly-selected HSAs in Balaka and Salima districts in Malawi to record pregnancies, births and deaths in their village health registers. The HSAs extracted these data every month and sent them to the districts, where the project coordinators forwarded them to the National Statistical Office. This method — having HSAs report on vital events in their catchment areas — was found on the basis of initial qualitative research to be feasible for implementation in these two districts in Malawi, and was welcomed by all stakeholders including the Ministry of Health.

During the first fifteen months of implementation (from January 2010 to March 2011), the method generated data that produced under-five mortality rates that were 84% of the mortality rates from the survey, and statistically equivalent to these rates based on a margin of 20%. In the same period, infant and neonatal mortality rates were respectively 70% and 65% of the survey rate, although still statistically equivalent to it. The level of under-estimation increased over time, suggesting that the approach, as implemented, was not stable and therefore could not be relied upon to produce accurate trends in childhood mortality. In terms of events reported, the results indicated that HSAs were more likely to miss neonatal and infant deaths than older deaths. No systematic patterns were observed by the sex of the child.

The RMM approach based on reporting of births and deaths at the community level was intentionally designed to build on and reinforce the existing government system for reporting vital events. This approach presents several advantages but also some challenges. In terms of advantages, the approach reinforces an existing government structure for monitoring of community vital events, and therefore avoids creating a separate parallel structure. This enhances the potential for sustainability by engaging the Ministry of Health's actors at district level. The RMM project benefited from strong support by the Ministry of Health (MOH) and the district health management teams. At district level, MOH staff, such as the regular HSA supervisors, were employed to support the reporting, thereby building all activities within the regular tasks conducted by these personnel at district level. In addition, the method used existing government registers that were already familiar to district staff and HSAs. Training, review meetings, and support were coordinated easily with district staff who participated fully in the activities. Furthermore, the presence of NSO on the ground, with its strong government credentials, contributed to the smooth running of the project in the two districts and facilitated access to the Environmental Health Officers responsible for supervision of the HSAs.

There were also numerous challenges in implementing the approach. Building the RMM project into an existing government structure has meant that shortcomings of the existing system also affected project operations. For example, RMM project staff had limited authority over the government MOH employees at district level who were responsible for implementation, and although all processes were discussed with the MOH and district officials, there remained a perception among district health management teams that RMM activities were not a routine part of their responsibilities. In addition, RMM project staff could not control turnover among the HSAs selected for participation in the project, their supervisors, or the district RMM coordinators. Although the district Environmental Health Officers made sincere efforts to replace RMM HSAs that transferred out or left, turnover among supervisors was not within their control. Ensuring regular and frequent supervision of the RMM HSAs was a constant challenge, despite the fact that the project provided transportation incentives and cell phone airtime to support contact between HSAs and their supervisors. Irregularities in supervision were exacerbated by the chronic fuel shortage that the country experienced during the project period. The rapid decline in the identification and reporting of vital events observed was partly due to a relaxation over the course of the study period in the supervision of the HSAs. The flow of completed forms from HSAs to supervisors and districts was problematic, and there were instances where completed forms were lost and the HSAs were required to re-extract the data from their VHRs.

Although the approach of using HSAs for monitoring child mortality appears in principle to be feasible and attractive, it requires high levels of inputs to ensure HSAs and their supervisors are well-trained, have continued support, and that incentives and supervision are provided continuously to maintain high levels of performance. The number of under-five deaths reported by HSAs went up in the months immediately following the training and provision of incentive packages, but dropped thereafter. It then peaked again following a review meeting that brought HSAs, supervisors, district management teams, and partners together to review the data and provide feedback. Ensuring stability of reporting required continued support and feedback to the HSAs. Recording of vital events in VHRs was already part of the job description of the HSAs prior to the implementation of RRT, but significant investments were needed to transform these formal terms of reference into reality. Lessons learnt from this first application are being used to improve and streamline the method in a second round of testing.

The analysis presented did not take in to account the possible effect of migration on HSA reporting of vital events. Although HSAs are trained to update their village health registers for transfers in and out of their catchment areas, it is unclear the extent to which this was systematically documented and taken into account in the reporting. In addition, the full birth histories collecting in the household survey may have been affected by recall errors, although we expect this bias was minimal because the assessment periods were recent.

Although this first test of the accuracy of vital events data reported by HSAs in Malawi for monitoring levels and trends in under-five mortality showed disappointing results, the approach has the potential to serve as a strategy for monitoring child mortality if it receives sufficient attention from the MOH. In addition, it can be easily linked to the development of a strong vital registration system, a current priority of the Malawi Government.

## Supporting Information

STROBE Checklist S1 (DOC)

Web Annex S1 HSA Data Extraction Form.
(DOCX)

Web Annex S2 Design, methods and results of the household mortality survey.
(DOCX)

Web Annex S3 Monthly number of births and neonatal, infant and under-five deaths reported by HSAs.
(DOCX)

## References

[1] UNICEF (2012) Levels and trends in child mortality. Estimates Developed by the UN Inter-agency Group for Child Mortality Estimation. New York, USA: UNICEF, WHO, The World Bank, United Nations.

[2] Lozano R, Wang H, Foreman KJ, Rajaratnam JK, Naghavi M, et al (2011) Progress towards Millennium Development Goals 4 and 5 on maternal and child mortality: an updated systematic analysis. Lancet 378: 1139–1165. 21937100 10.1016/S0140-6736(11)61337-8

[3] Requejo J, Bryce J, Victora CG, Countdown Technical Working Groups (2012) Countdown to 2015: Building a Future for Women and Children. New York, USA: WHO and UNICEF.

[4] Mathers C, Boerma T (2010) Mortality measurement matters: improving data collection and estimation methods for child and adult mortality. PLoS Med 7: e1000265. 20405053 10.1371/journal.pmed.1000265 PMC2854121

[5] Setel PW, Macfarlane SB, Szreter S, Mikkelsen L, Jha P, et al (2007) A scandal of invisibility: making everyone count by counting everyone. Lancet 370: 1569–1577. 17992727 10.1016/S0140-6736(07)61307-5

[6] Mahapatra P, Shibuya K, Lopez AD, Coullare F, Notzon FC, et al (2007) Civil registration systems and vital statistics: successes and missed opportunities. The Lancet 370: 1653–1663. 10.1016/S0140-6736(07)61308-7 18029006

[7] Arudo J, Gimnig JE, Ter Kuile FO, Kachur SP, Slutsker L, et al (2003) Comparison of governement statistics and demographic surveillance to monitor mortality in children less than five years old in rural Western Kenya. Am J Trop Med Hyg 68: 30–37. 12749483

[8] AbouZahr C, Boerma JT (2005) Health information systems: the foundations of public health. Bulletin of the World Health Organization 83: 578–583. 16184276 PMC2626318

[9] Korenromp EL, Arnold F, Williams BG, Nahlen BL, Snow RW (2004) Monitoring trends in under-5 mortality rates through national birth history surveys. Int J Epidemiol 33: 1293–1301. 15319411 10.1093/ije/dyh182

[10] Commission on Information and Accountability for Women's and Children's Health (2011) Keeping promises, measuring results. Geneva, Switzerland: WHO.

[11] Edward A, Ernst P, Taylor C, Becker S, Mazive E, et al (2007) Examining the evidence of under-five mortality reduction in a community-based programme in Gaza, Mozambique. Trans R Soc Trop Med Hyg 101: 814–822. 17482222 10.1016/j.trstmh.2007.02.025

[12] The Institute for International Programs (2007) Expert consultation on methodological alternatives for monitoring child mortality. Consultation Report. Baltimore, Maryland, USA: Johns Hopkins Bloomberg School of Public Health.

[13] National Statistical Office (NSO) (2009) Population and Housing Census 2008. Main census report. Zomba, Malawi: National Statistical Office.

[14] National Statistical Office (NSO), ICF Macro (2011) Malawi Demographic and Health Survey 2010. Zomba, Malawi, and Calverton, Maryland, USA.

[15] Nsona H, Mtimuni A, Daelmans B, Callaghan-Koru JA, Gilroy K, et al (2012) Scaling up integrated community case management of childhood illness: update from Malawi. Am J Trop Med Hyg 87: 54–60. 23136278 10.4269/ajtmh.2012.11-0759 PMC3748522

[16] Ministry of Health (2008) Health Surveillance Assistants training curriculum. Lilongwe, Malawi: Ministry of Health.

[17] Kock MC, Muula AS (2013) Motivation and job satisfaction of Health Surveillance Assistants in Mwanza, Malawi: an explorative study. Malawi Medical Journal 25: 5–11. 23717748 PMC3653191

[18] Rosner B (2000) Fundamentals of Biostatistics. Pacific Grove: Duxbury. p792.

[19] International Programs Center for Technical Assistance (2013) CSPro User' guide. Version 5.0.3. Washington DC: Population Division, US Census Bureau.

[20] StataCorp (2011) Stata: Release 12. Statistical Software. College Station: StataCorp LP.

[21] Lohr SL (1999) Sampling: design and analysis. California: Duxbury Press. p489.

