# Supplementary material for: Monitoring Child Mortality through Community Health Worker Reporting of Births and Deaths in Malawi: Validation against a Household Mortality Survey
Source: PLoS One. 2014 Feb 18;9(2):e88939. doi: 10.1371/journal.pone.0088939 (PMC3928330; doi:10.1371/journal.pone.0088939)
Supplement: STROBE Checklist S1 — (DOC) [file pone.0088939.s001.doc]

STROBE Statement—Checklist of items that should be included in reports of ***cross-sectional studies***

**Monitoring Child Mortality through Community Health Worker Reporting of Births and Deaths:**

**Validation against a Cross-Sectional Household Survey with Full Birth History**

|  | Item No | Recommendation |  | Comment from authors |  |
| --- | --- | --- | --- | --- | --- |
| **Title and abstract** | 1 | (*a*) Indicate the study’s design with a commonly used term in the title or the abstract |  | We chose a title that reflects a combination of prospective reporting and a cross sectional survey |  |
|  |  | (*b*) Provide in the abstract an informative and balanced summary of what was done and what was found |  | The abstract includes succinct description of the study background, objective, methods, the results, and conclusions |  |
| Introduction | | |  |  |  |
| Background/rationale | 2 | Explain the scientific background and rationale for the investigation being reported |  | Detailed scientific background and rationale of the study globally and in the context of Malawi are provided. |  |
| Objectives | 3 | State specific objectives, including any prespecified hypotheses |  | We state the main objective of the study and the main analytical procedure and our margin of equivalence in the methods tested |  |
| Methods | | |  |  |  |
| Study design | 4 | Present key elements of study design early in the paper |  | We provide detailed description of the implementation of the project and the analytical procedures for validation of the mortality results in the method section |  |
| Setting | 5 | Describe the setting, locations, and relevant dates, including periods of recruitment, exposure, follow-up, and data collection |  | We provide a summary description of Malawi in the introduction and a description of the two study districts in the method section. |  |
| Participants | 6 | (*a*) Give the eligibility criteria, and the sources and methods of selection of participants |  | We provide description of participant eligibility to the gold-standard mortality survey in the method section |  |
| Variables | 7 | Clearly define all outcomes, exposures, predictors, potential confounders, and effect modifiers. Give diagnostic criteria, if applicable |  | The main variables in the study are the neonatal, infant and under-five mortality, all explained in the method section. |  |
| Data sources/ measurement | 8* | For each variable of interest, give sources of data and details of methods of assessment (measurement). Describe comparability of assessment methods if there is more than one group |  | We provide description of various sources of data used. |  |
| Bias | 9 | Describe any efforts to address potential sources of bias |  | We acknowledged that the analysis did not adjust for migration effects or possible recall bias in the full birth history |  |
| Study size | 10 | Explain how the study size was arrived at |  | We described in the method section how sample size was computed |  |
| Quantitative variables | 11 | Explain how quantitative variables were handled in the analyses. If applicable, describe which groupings were chosen and why |  | Not applicable |  |
| Statistical methods | 12 | (*a*) Describe all statistical methods, including those used to control for confounding |  | We describe the computation procedures for the mortality rates and associated standard errors. |  |
|  |  | (*b*) Describe any methods used to examine subgroups and interactions |  | Not applicable |  |
|  |  | (*c*) Explain how missing data were addressed |  | We explain in the analysis section, how missing was data was handled in both the HSA data |  |
|  |  | (*d*) If applicable, describe analytical methods taking account of sampling strategy |  | We described that analysis using the survey data took into account the sampling weight |  |
|  |  | (*e*) Describe any sensitivity analyses |  | We did not conduct any sensitivity analysis |  |
| Results | | |  |  |  |
| Participants | 13* | (a) Report numbers of individuals at each stage of study—eg numbers potentially eligible, examined for eligibility, confirmed eligible, included in the study, completing follow-up, and analysed |  | We provide supporting information on numbers of births and deaths from HSA data and additional description and quality assessment of the mortality data from the full birth history |  |
|  |  | (b) Give reasons for non-participation at each stage |  | Not applicable |  |
|  |  | (c) Consider use of a flow diagram |  | We do not think a flow chart would be informative for this study |  |
| Descriptive data | 14* | (a) Give characteristics of study participants (eg demographic, clinical, social) and information on exposures and potential confounders |  | This was not relevant for our study |  |
|  |  | (b) Indicate number of participants with missing data for each variable of interest |  | We presented a figure showing the number of HSAs that reported data by month. In addition, we present response rate for the survey in the webannex 2 (in supporting information) |  |
| Outcome data | 15* | Report numbers of outcome events or summary measures |  | We reported number of deaths and births and neonatal, infant and under-five mortality rates |  |
| Main results | 16 | (*a*) Give unadjusted estimates and, if applicable, confounder-adjusted estimates and their precision (eg, 95% confidence interval). Make clear which confounders were adjusted for and why they were included |  | We provide 95% confidence intervals for the mortality rates and ratios computed.  No other adjustment was needed in our analysis |  |
|  |  | (*b*) Report category boundaries when continuous variables were categorized |  | Not applicable |  |
|  |  | (*c*) If relevant, consider translating estimates of relative risk into absolute risk for a meaningful time period |  | Not applicable |  |
| Other analyses | 17 | Report other analyses done—eg analyses of subgroups and interactions, and sensitivity analyses |  | Not done |  |
| Discussion | | |  |  |  |
| Key results | 18 | Summarise key results with reference to study objectives |  | The discussion section starts with a summary of the study objectives and the results |  |
| Limitations | 19 | Discuss limitations of the study, taking into account sources of potential bias or imprecision. Discuss both direction and magnitude of any potential bias |  | We provide detail discussion of advantages and challenges of the project and assess the limitations of the analysis |  |
| Interpretation | 20 | Give a cautious overall interpretation of results considering objectives, limitations, multiplicity of analyses, results from similar studies, and other relevant evidence |  | The discussion provides an interpretation of the main results |  |
| Generalisability | 21 | Discuss the generalisability (external validity) of the study results |  | Discussed |  |
| Other information | | |  |  |  |
| Funding | 22 | Give the source of funding and the role of the funders for the present study and, if applicable, for the original study on which the present article is based |  | We included a section of funding at end of the manuscript |  |

*Give information separately for exposed and unexposed groups.

**Note:** An Explanation and Elaboration article discusses each checklist item and gives methodological background and published examples of transparent reporting. The STROBE checklist is best used in conjunction with this article (freely available on the Web sites of PLoS Medicine at http://www.plosmedicine.org/, Annals of Internal Medicine at http://www.annals.org/, and Epidemiology at http://www.epidem.com/). Information on the STROBE Initiative is available at www.strobe-statement.org.
