# Supplementary material for: Monitoring Child Mortality through Community Health Worker Reporting of Births and Deaths in Malawi: Validation against a Household Mortality Survey
Source: PLoS One. 2014 Feb 18;9(2):e88939. doi: 10.1371/journal.pone.0088939 (PMC3928330; doi:10.1371/journal.pone.0088939)
Supplement: Web Annex S1 — HSA Data Extraction Form. (DOCX) [file pone.0088939.s002.docx]

**Web Annex 1**

**HSA Data Extraction Form**

| **DISTRICT**: ………………………………………………..  **EVALUATION OF MNCH RAPID SCALE UP IN MALAWI**  **NATIONAL STATISTICAL OFFICE- JOHNS HOPKINS UNIVERSITY**  **MONTHLY HSA RECORDING OF VITAL EVENTS EXTRACTION FORM** | **TA/STA**: ………………………………………………… |
| --- | --- |
| **NAME OF PLACE/GVH**: ……………………………………………….. | **HEALTH FACILITY CODE**:………………………….. |
| **NAME OF HSA**: ……………………………………………….. | **HSA CATCHMENT CODE:_** _ _ _ _ _ |
| **MONTH & YEAR**: _ _ / _ _ _ _ |  |

**SCHEDULE B: PREGNANCIES**

| **LINE NO**  **(B01)** | **CODE NO OF WOMAN FROM VILLAGE HEALTH REGISTER**  **(B02)** | **AGE**  **(B03)** | **LMP (month & year)**  **(B04)** | **GESTATION IN MONTHS**  **(B05)** | **COMENT IF NECESSARY**  **(BO5)** |
| --- | --- | --- | --- | --- | --- |
| 1 |  |  |  |  |  |
| 2 |  |  |  |  |  |
| 3 |  |  |  |  |  |
| 4 |  |  |  |  |  |
| 5 |  |  |  |  |  |
| 6 |  |  |  |  |  |
| 7 |  |  |  |  |  |
| 8 |  |  |  |  |  |
| 9 |  |  |  |  |  |
| 10 |  |  |  |  |  |
| 11 |  |  |  |  |  |
| 12 |  |  |  |  |  |
| 13 |  |  |  |  |  |
| 14 |  |  |  |  |  |
| 15 |  |  |  |  |  |
| 16 |  |  |  |  |  |
| 17 |  |  |  |  |  |
| 18 |  |  |  |  |  |
| 19 |  |  |  |  |  |
| 20 |  |  |  |  |  |
| 21 |  |  |  |  |  |
| 22 |  |  |  |  |  |
| 23 |  |  |  |  |  |
| 24 |  |  |  |  |  |
| 25 |  |  |  |  |  |
| 26 |  |  |  |  |  |
| 27 |  |  |  |  |  |
| 28 |  |  |  |  |  |
| 29 |  |  |  |  |  |
| 30 |  |  |  |  |  |
| 31 |  |  |  |  |  |
| 32 |  |  |  |  |  |
| 33 |  |  |  |  |  |
| 34 |  |  |  |  |  |
| 35 |  |  |  |  |  |
| 36 |  |  |  |  |  |
| 37 |  |  |  |  |  |
| 38 |  |  |  |  |  |
| 39 |  |  |  |  |  |
| 40 |  |  |  |  |  |

**SCHEDULE C: BIRTHS, *Record only live births***

| **LINE NO**  **(C01)** | **CODE NO OF CHILD MOTHER FROM VILLAGE HEALTH REGISTER**  **(C02)** | **SEX OF CHILD**  **(C03)** | **DATE OF BIRTH**  **DD/MM/YYYY**  **(C04)** | **PLACE OF BIRTH**  **Home=1**  **TBA=2**  **H/C=3**  **Hospital=4**  **(C05)** | **COMENT IF NECESSARY** |
| --- | --- | --- | --- | --- | --- |
| 1 |  | M F | _ _ / _ _ / _ _ _ _ |  |  |
| 2 |  | M F | _ _ / _ _ / _ _ _ _ |  |  |
| 3 |  | M F | _ _ / _ _ / _ _ _ _ |  |  |
| 4 |  | M F | _ _ / _ _ / _ _ _ _ |  |  |
| 5 |  | MF | _ _ / _ _ / _ _ _ _ |  |  |
| 6 |  | M F | _ _ / _ _ / _ _ _ _ |  |  |
| 7 |  | M F | __ / _ _ / _ _ _ _ |  |  |
| 8 |  | M F | _ _ / _ _ / _ _ _ _ |  |  |
| 9 |  | M F | _ _ / _ _ / _ _ _ _ |  |  |
| 10 |  | M F | _ _ / _ _ / _ _ _ _ |  |  |
| 11 |  | M F | _ _ / _ _ / _ _ _ _ |  |  |

FORM HSA2

**SCHEDULE D: DEATHS**

| **LINE NO** | **CODE NO OF PERSON FROM VILLAGE HEALTH REGISTER** | **DATE OF BIRTH**  **DD/MM/YYYY** | **SEX** | **DATE OF DEATH**  **DD/MM/YYYY** | **AGE*** | **AGE GROUP**  **< 1yr = 1**  **1-4yrs = 2**  **≥ 5yrs = 3** | **PLACE OF DEATH**  ***(If health facility, write name*)** | **REPORTED CAUSE OF DEATH** | ***NOTE: ASK THE FOLLOWING FOR DEATHS OF WOMEN AGED 12 YEARS + TO PROBE IF MATERNAL DEATH*** | | |
| --- | --- | --- | --- | --- | --- | --- | --- | --- | --- | --- | --- |
|  |  |  |  |  |  |  |  |  | **Was (PERSON) Pregnant when she died?**  **Yes=1**  **No=2** | **Did (PERSON) die during Childbirth?**  **Yes=1**  **No=2** | **Did (PERSON) die within 2 months after delivery?**  **Yes=1**  **No=2** |
| **(D01)** | **(D02)** | **(D03)** | **(D04)** | **(D05)** | **(D06)** | **(D07)** | **(D08)** | **(D09)** | **(D10)** | **(D11)** | **(D12)** |
| 1 |  | _ _ / _ _ / _ _ _ _ | M F | _ _ / _ _ / _ _ _ _ |  |  |  |  |  |  |  |
| 2 |  | _ _ / _ _ / _ _ _ _ | M F | _ _ / _ _ / _ _ _ _ |  |  |  |  |  |  |  |
| 3 |  | _ _ / _ _ / _ _ _ _ | M F | _ _ / _ _ / _ _ _ _ |  |  |  |  |  |  |  |
| 4 |  | _ _ / _ _ / _ _ _ _ | M F | _ _ / _ _ / _ _ _ _ |  |  |  |  |  |  |  |
| **5** |  | _ _ / _ _ / _ _ _ _ | M F | _ _ / _ _ / _ _ _ _ |  |  |  |  |  |  |  |

** Include age at death in days if less than one month, in months if less than two years and in years if more than two years. In case age at death is in days or in months, please write days or months in brackets.*

| **CHECK ED BY; SUPERVISOR NAME; SIGNATURE; DATE;** |
| --- |

| \|  \| \| --- \| |  |
| --- | --- | --- |

FORM HSA2
