# Supplementary material for: Monitoring Child Mortality through Community Health Worker Reporting of Births and Deaths in Malawi: Validation against a Household Mortality Survey
Source: PLoS One. 2014 Feb 18;9(2):e88939. doi: 10.1371/journal.pone.0088939 (PMC3928330; doi:10.1371/journal.pone.0088939)
Supplement: Web Annex S2 — Design, methods and results of the household mortality survey. (DOCX) [file pone.0088939.s003.docx]

# Web Annex 2

# Design, methods and results of the “gold-standard” mortality survey

The aim of the RRT project was to provide rigorous validations of promising approaches, as a basis for making decisions about which methods justify broader application within Malawi and in other, similar settings. The best available method for measuring under-five mortality was the collection of full birth histories from women of reproductive age in high-quality household surveys conducted using the methods developed under the Demographic and Health Survey program supported by USAID. IIP-JHU worked in collaboration with the Malawi NSO to design and carry out such a survey in two RRT districts in Malawi. The survey was designed to serve as the gold standard against which the data generated through RRT methods could be validated, and thus, included rigorous quality control measures.

**B.1. Objective**

The main objective of the household mortality survey was to obtain the data needed to validate the three RRT approaches implemented in two districts in Malawi. More specifically the survey was designed to:

1. Estimate childhood mortality in the districts of Balaka and Salima, based on women’s report of their full birth history; and
2. Support validation of the RRT methods implemented in Balaka and Salima.

**B.2. Methods**

**B.2.1. Sample size**

The survey collected data from 24,000 households distributed equally between the two RRT districts. Section 8.1.2 above provides details of the sample size calculation.

**B.2.2. Sampling frame and sample selection**

The 2008 Population Census frame was used to select the primary sampling units or enumeration areas (EA) for the survey, with probability proportionate to size. The sampling was stratified by district.

The plan was to select 35 households per EA, making a total of 686 EAs, equally divided across the two districts. Thus, 343 EAs were selected in each district. In Balaka, the total number of EAs in the entire district was 294. However, there were large variations in the size of EAs, ranging from 21 to 650 households with an average number of 253 households. To reach the 343 EAs needed in the sample, large EAs with more than 330 households were divided into two or three EAs, and all resulting EAs in the district were included in the sample (now a census of EAs). All households within each EA were listed and a sample of 35 households was selected using a systematic random sampling procedure. Thus, in Balaka, only a single stage sample of households was conducted.

In Salima, the total number of EAs is 435. Two-stage sampling was used, with EA selection at first stage and household selection at a second stage. The primary stage selection was conducted using a systematic random sampling procedure with probability proportionate to size in terms of households. The size of EAs varied from 15 to 780 households. During the sample selection, some very large EAs were selected twice; one EA was selected three times and another four times. These EAs were divided into the number of times they were selected, and each segment used a separate cluster. In addition, very small EAs were combined with neighboring EAs before the random selection was completed. All households in each selected EAs were listed and a random selection of 35 households occurred using systematic random sampling procedures.

Given that the same number of households was selected in each cluster, the probability of selection of a household was not the same for every home included in the sample. Thus, sampling weights were computed and used during analysis to ensure the representativeness of the results.

**B.2.3. Questionnaires**

These authors developed a structured, pre-coded questionnaire adapted from the standard DHS tool. The household form collected basic information about each household member as well as gathering information about access to water and sanitation facilities, roof, floor and wall construction materials, number of sleeping rooms, possession of land and livestock ownership and household ownership of assets such as mobile phones, bicycles, radios, and others items. GPS coordinates were collected for each household.

The “individual woman” form was administered to all women ages 15-49 living within the household. Women were asked to provide age, marital status, and educational level. They were asked a summary list of questions on whether they had ever given birth, and if so, how many of their children were still alive at the time of the interview. If a woman reported having children, a full birth history module was administered that included detailed questions about each child.

All questions were written in both English and the local language of Chichewa to assist the interviewers, but interviews were conducted in Chichewa most often. The tool was pre-tested during interviewer field practice sessions in rural villages near Zomba in October 2011. To ensure the highest possible survey quality, the questionnaire was kept short and administration took 15 to 30 minutes.

**B.2.4. Recruitment and training of fieldworkers**

The Human Resources Department of the NSO assisted by technical staff (survey managers) led the process of recruiting field staff. This involved advertising, short listing, interviewing, reviewing candidates’ performance in the written skill test, and selecting successful candidates.

In August 2011, advertisements for the post of field staff was made in local newspapers, calling for would be candidates to fill the post of survey enumerators. The required credentials included possession of a Malawi School Certificate of Education (MSCE) with credits in Mathematics and English; an equivalent of the British O Level certificate, and experience in household surveys and specifically the Malawi Demographic and Health Survey (MDHS). The advertisements were maintained for three weeks.

Short listing commenced immediately upon receipt of applications and potential interviewees were selected based on the standard criteria listed above. The interview was primarily skill-based with each candidate presented with a written case study and asked to complete the RRT survey questionnaire in writing. The completed questionnaires were marked by survey managers, with special attention to whether the candidate demonstrated a correct understanding of the questionnaire. Successful candidates were called for training one week later.

Training commenced in 10 October 2011 and continued for eight days, including two days of supervised field practice. Training methods included classroom training on each questionnaire module, mock interviews, plenary discussions, and supervised field practice. The training explained how to complete the household questionnaire, including the information panel, the listing of household members and their characteristics and information on water and sanitation. The individual woman’s questionnaire included questions pertaining to her characteristics and the summary of full birth histories. Other sessions focused on how to conduct a successful interview, the roles, and responsibilities of field staff, mapping using a global positioning system (GPS), sampling, household listing, and checking and editing competed questionnaires. Field practice sessions focused on improving field staff capability to locate households, conduct interviews, and edit questionnaires. Plenary sessions were held at the end of each field practice exercise to discuss the experiences of field staff and exchange knowledge as well as to fine-tune weak areas observed by field practice supervisors. A final test was conducted and used to select the best-performing trainees, identify supervisors, and for forming well-balanced field teams.

**B.2.5. Fieldwork**

Fieldwork started on 24 October 2011 and ended on 17 February 2012. A survey coordinator was appointed for each district, along with eight survey teams composed of one supervisor, five enumerators and a driver, with 16 survey teams. At district level, the survey coordinator ensured that all responsible district authorities (District commissioner, Officer in Charge at Police, Traditional leaders) were notified about the survey and its entire organization (the teams, tasks, location, and timing of data collection).

The survey coordinators were the link between the field staff on the ground and the survey coordinating personnel at the NSO headquarters. Each survey coordinator was responsible for logistical and technical backstopping to the teams. This involved ensuring availability of logistical supplies such as fuel for the vehicles, questionnaires, listing forms, maps, chalk, and every other equipment and consumables required during fieldwork, throughout the survey period.

At the team level, a supervisor was responsible for coordination of field workers’ daily assignments and the listing, interviewing, and editing of questionnaires. The teams began in each EA by first listing all the households. Then the team supervisor randomly sampled 35 households and the interviews commenced immediately.

The team supervisor ensured that a daily schedule was prepared, outlining the tasks to be done, the location where the task was to be performed, and the team responsible for the task. The team leader also ensured that all teams had the necessary supplies. At the end of each field day, interviewers edited questionnaires before submitting them to their team leader, who also reviewed each questionnaire, conducted callbacks, and re-interviewed as needed.

Four senior survey supervisors from NSO visited field teams on a rotating basis throughout the survey period, conducting on-the-spot supervision. Key areas of focus during these visits were checking whether mapping was correct, observing how interviews were conducted, assessing data quality, and providing on-the-spot corrections. Upon their return to NSO, any serious anomalies were discussed at the central coordinating office with the survey senior management team. Decisions were communicated to responsible field personnel immediately. Periodic supervision reports at all levels of survey coordination and implementation were produced and provided further input for improving survey management.

**B.2.6. Quality Control**

A number of quality control mechanisms were put in place. During fieldwork, team leaders were required to observe at least one interview from each interviewer in a cluster. They were also required to conduct a re-interview in one household for each interviewer.

After completion of the interviews, the team leader checked all the completed forms for inconsistencies and signed the edited questionnaires that were satisfactory. Questionnaires with inconsistencies were sent back to the interviewers for correction. Guidelines for questionnaire editing were detailed in the Supervisors’ Manual for the survey. At district level, a field coordinator spot-checked all completed questionnaires and initialed them for onward transfer to NSO. When the forms arrived at the NSO, four office editors scrutinized the questionnaires for any inconsistencies before sending them on to the data entry unit at NSO.

**B.2.6. Data entry and processing**

All completed data forms were packed in A3 envelopes with a dispatch form, a listing form, and the Interviewer and Supervisor assignment sheets. This packet was sent to the NSO for data processing. Upon arrival at the NSO, forms were registered in a questionnaire registry inventory file and spreadsheet. Four office editors were trained to check the questionnaires for any inconsistencies using prescribed data editing guidelines.

Once the editing was completed, questionnaires from each cluster were passed on to the assigned data entry clerk. A CSPro 4.1 data application program was developed that allowed for double data entry. Sixteen data entry clerks were trained on data processing procedures. Once data for a cluster were entered, the forms were passed to a second data entry clerk for double verification to eliminate entry errors. The two datasets were then checked and reconciled.

Completed data sets were then transferred to STATA for further cleaning. All data collected on GPS were copied by the appropriate software and stored in a separate file. All data analyses were conducted in STATA.

**B.2.7. Survey results**

The survey successfully reached a household response rate of 98.5%, 99.1% in Balaka and 97.9% in Salima. Eligible women response rates were estimated at 96.3% (95.7% in Balaka and 97.9% in Salima). These rates are comparable to the 2010 DHS rates.

**Table B1**: Results of households and women’s interviews

| **Result** |  | **District** | |  |  |
| --- | --- | --- | --- | --- | --- |
|  |  | **Balaka** | **Salima** |  | **Total** |
|  |  |  |  |  |  |
| Sample households |  | 12005 | 12005 |  | 24010 |
| Interviewed households |  | 11,899 | 11,749 |  | 23,648 |
|  |  |  |  |  |  |
| **Households response rate** |  | **99.1** | **97.9** |  | **98.5** |
|  |  |  |  |  |  |
| Eligible women in interviewed households |  | 10,787 | 11,117 |  | 21,904 |
| Women interviewed |  | 10,322 | 10,763 |  | 21,085 |
|  |  |  |  |  |  |
| **Women's response rate** |  | **95.7** | **96.8** |  | **96.3** |

**B.2.7. Household population in Balaka and Salima**

**Table B2:** Percent distribution of the de facto household population by five-year age groups, according to sex and district

| Age group |  | Total | | |  | Balaka | | |  | Salima | | |
| --- | --- | --- | --- | --- | --- | --- | --- | --- | --- | --- | --- | --- |
|  |  | Males | Females | Total |  | Males | Females | Total |  | Males | Females | Total |
| 0-4 |  | 18.6 | 18.0 | 18.3 |  | 18.2 | 18.0 | 18.1 |  | 19.0 | 17.9 | 18.5 |
| 5-9 |  | 17.8 | 16.9 | 17.3 |  | 18.0 | 16.8 | 17.3 |  | 17.5 | 17.0 | 17.3 |
| 10-14 |  | 14.6 | 14.0 | 14.3 |  | 14.7 | 14.0 | 14.3 |  | 14.5 | 14.1 | 14.3 |
| 15-19 |  | 10.3 | 9.4 | 9.8 |  | 10.4 | 9.2 | 9.8 |  | 10.1 | 9.7 | 9.9 |
| 20-24 |  | 7.2 | 8.5 | 7.8 |  | 7.3 | 8.4 | 7.9 |  | 7.1 | 8.5 | 7.8 |
| 25-29 |  | 7.0 | 7.7 | 7.3 |  | 6.8 | 7.6 | 7.2 |  | 7.2 | 7.8 | 7.5 |
| 30-34 |  | 5.6 | 5.5 | 5.6 |  | 5.5 | 5.5 | 5.5 |  | 5.6 | 5.6 | 5.6 |
| 35-39 |  | 4.9 | 4.2 | 4.5 |  | 4.7 | 4.0 | 4.3 |  | 5.1 | 4.5 | 4.8 |
| 40-44 |  | 3.2 | 2.6 | 2.9 |  | 3.0 | 2.7 | 2.9 |  | 3.4 | 2.5 | 2.9 |
| 45-49 |  | 2.4 | 2.1 | 2.3 |  | 2.4 | 2.2 | 2.3 |  | 2.5 | 2.0 | 2.3 |
| 50-54 |  | 1.9 | 3.1 | 2.5 |  | 2.0 | 3.3 | 2.7 |  | 1.9 | 2.9 | 2.4 |
| 55-59 |  | 1.7 | 2.0 | 1.9 |  | 1.6 | 2.1 | 1.9 |  | 1.7 | 2.0 | 1.8 |
| 60-64 |  | 1.9 | 2.0 | 1.9 |  | 2.1 | 2.2 | 2.1 |  | 1.6 | 1.7 | 1.7 |
| 65-69 |  | 1.1 | 1.3 | 1.2 |  | 1.1 | 1.2 | 1.2 |  | 1.0 | 1.3 | 1.2 |
| 70-74 |  | 0.8 | 1.1 | 1.0 |  | 0.9 | 1.1 | 1.0 |  | 0.8 | 1.0 | 0.9 |
| 75-79 |  | 0.5 | 0.7 | 0.6 |  | 0.5 | 0.8 | 0.7 |  | 0.5 | 0.6 | 0.6 |
| 80+ |  | 0.7 | 0.9 | 0.8 |  | 0.8 | 0.9 | 0.9 |  | 0.5 | 0.8 | 0.7 |
| Missing |  | 0.0 | 0.0 | 0.0 |  | 0.0 | 0.0 | 0.0 |  | 0.0 | 0.0 | 0.0 |
|  |  |  |  |  |  |  |  |  |  |  |  |  |
| Total |  | 100.0 | 100.0 | 100.0 |  | 100.0 | 100.0 | 100.0 |  | 100.0 | 100.0 | 100.0 |
| Number |  | 52,039 | 54,648 | 106,687 |  | 25,388 | 27,257 | 52,645 |  | 26,701 | 27,341 | 54,042 |

**Figure B1**: Population age pyramid for Balaka and Salima


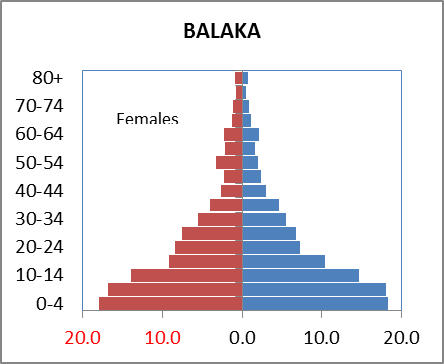

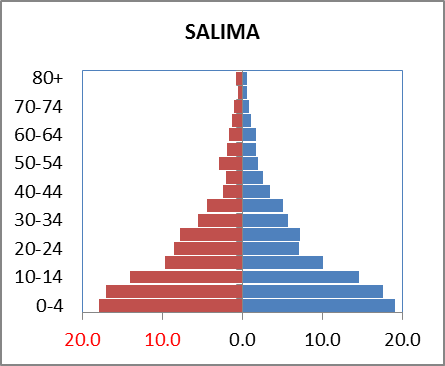

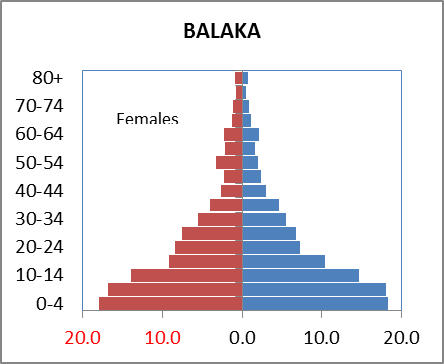

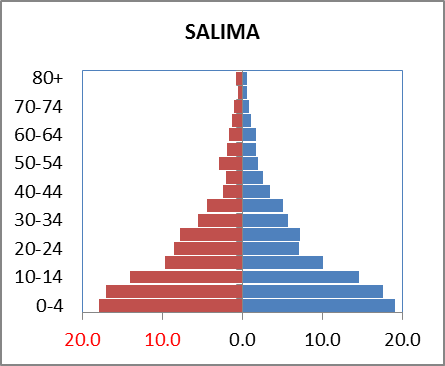


Males

Females

Males

**B.2.7. Data quality assessment**

This team conducted preliminary internal quality assessment of the data by examining the age and date of birth. A more extensive quality assessment is currently ongoing.

**a) Transfer of eligible females outside eligibility age range**

Figure B1 shows the age distribution of females aged 9 to 54 years in the sampled households. In both districts, there are drops in the number of female household residents between the ages of 12 and 15, and increases at ages 50-52, suggesting some transference of eligible women outside the age eligibility range. This phenomenon is characteristic of household surveys where interviewers are required to identify women of reproductive age in the households for further interview. Figure B1 compare the age distribution of females to that from the DHS 2010 data. The age distribution is very similar.

**Figure B1:** Distribution of household females by age and by districts (only age 9-54 years are shown)

**b) Births by month and year**

Figures B2 and B3 show the distribution of births by month and year from 2001 to 2011 for Balaka and Salima districts, respectively. The patterns look generally similar across years. In Salima, there is a consistent reduction in the number of births reported across years. In both districts, the bottom curve (in red) indicates the patterns in 2002 when the country experienced a severe famine. The patterns of distribution of births in Balaka and Salima are also similar to the nationwide patterns observed in data from the DHS 2010 (figure B4).

**Figure B2:** Distribution of births by month and year of birth in Balaka

**Figure B3:** Distribution of births by month and year of birth in Salima

**Figure B4:** Distribution of births by month and year of birth Malawi, DHS 2010

**c) Sex ratio at birth**

The sex ratio at birth in normal populations is generally between 102 to 107 males per 100 females. Large departures from this range may suggest differential omission of births by sex. Figures B5, B6 and B7 show sex ratios at delivery for all births, living children, and children who died. Sex ratios at delivery for all births and for living children do not indicate any severe irregularities and compare fairly well with the similar sex ratios from DHS 2010. However, sex ratios for children who have died show a large dip in 1993 and prominent peaks in 1995/97. These may be indicative of specific contextual factors that will need further documentation. These patterns are reflected in the 2010 DHS data although the trends in the DHS 2010 data are not as extreme.

**d) Age at death**

Errors in age declaration can affect mortality estimation. Figures B7 and B8 show the distribution of neonatal deaths by age in days and the per month distribution of children who died between the ages of one and 24 months by age at death. The observed distributions are typical of empirical surveys and the tendency for deaths to “heap” at certain ages. For age at death for neonates, these data show heaping at ages one, seven, and 14 days. For children who died between the ages of one and 23 months, the distribution shows stronger heaping at age 12 months. This particular pattern would lead to an under-estimation of the infant mortality rate and an over-estimation of the child mortality rate. The under-five mortality rate will not be affected.

**Figure B5:** Sex Ratio at birth for births since 1990

**Figure B6**: Sex-ratio at birth for living children

**Figure B7:** Sex-ratio at birth for children who have died

**Figure B8**: Sex-ratio at birth for all, living and dead children, DHS 2010

**Figure B7:** Distribution of neonatal deaths by age at death in days (births since 1990)

**Figure B8:** Distribution of children who died between ages of 1 month and 24 months by age at death in month (births since 1990)

**e) Age patterns of deaths: ratio of early neonatal to neonatal deaths and neonatal deaths to infant deaths**

Table B3 presents the ratios of early neonatal to neonatal deaths and the ratio of neonatal to under-five deaths for the RRT and the 2010 DHS surveys. The ratio of early neonatal to neonatal deaths is generally within the range of 0.70 to 0.80. The ratios presented for the RRT survey in either Balaka or Salima are in the same range. However, compared to the DHS 2010, there is difference between the ratio in Salima. The DHS ratio is on the lower side suggesting an under-reporting of early neonatal deaths in the DHS.

The ratio of neonatal to under-five death is in the range of 0.30 in Balaka or Salima and is comparable to the DHS ratio.

**Table B3:** Ratio of early neonatal to neonatal deaths and ratio of neonatal to under-five deaths from the RRT survey and the 2010 DHS (all under-five deaths).

| **District** | **Ratio of early neonatal to neonatal deaths** | | **Ratio of neonatal to under-five deaths** | |
| --- | --- | --- | --- | --- |
|  | **RRT Survey** | **DHS 2010** | **RRT Survey** | **DHS 2010** |
| **Balaka** | 0.7511 | 0.7407 | 0.3144 | 0.2796 |
| **Salima** | 0.7171 | 0.6365 | 0.2759 | 0.3007 |
| **Total Balaka and Salima** | 0.7359 | 0.6784 | 0.2959 | 0.2918 |
| **Total Malawi** | na | 0.7207 | na | 0.2603 |

**B.3. Mortality results**

Figure B9 presents the trends in under-five mortality rate for Balaka, Salima, and for both districts combined. It shows two main features. First, it suggests that levels and trends in under-five mortality rates were similar in both districts. Second, under-five mortality appears to have declined sharply in these two districts between 2000 and 2006, and stalled thereafter at around 100 deaths per 1000 live births.

**Figure B9:** Trends in under-five mortality rates for Balaka, Salima and total

**Figure B10:** Trends in neonatal mortality rates for Balaka, Salima and total

**Table 10:** Neonatal (NNMR), post-neonatal (PNNMR), infant (IMR), child (CMR) and under-five (U5MR) mortality rates by year some 2000 to 2011 for Balaka, Salima and total

| Year |  | Balaka | | | | |  | Salima | | | | |  | Total | | | | |
| --- | --- | --- | --- | --- | --- | --- | --- | --- | --- | --- | --- | --- | --- | --- | --- | --- | --- | --- |
|  |  | NNMR | PNNMR | IMR | CMR | U5MR |  | NNMR | PNNMR | IMR | CMR | U5MR |  | NNMR | PNNMR | IMR | CMR | U5MR |
| 2011 |  | 41.3 | 23.6 | 64.9 | 25.9 | 89.1 |  | 32.7 | 28.6 | 61.3 | 34.5 | 93.7 |  | 37.1 | 26.0 | 63.1 | 30.1 | 91.3 |
| 2010 |  | 34.0 | 33.6 | 67.7 | 36.8 | 101.9 |  | 43.7 | 27.5 | 71.1 | 42.3 | 110.4 |  | 38.6 | 30.7 | 69.3 | 39.4 | 105.9 |
| 2009 |  | 37.2 | 25.0 | 62.2 | 38.2 | 98.0 |  | 38.8 | 31.0 | 69.8 | 51.4 | 117.6 |  | 38.0 | 28.0 | 65.9 | 44.3 | 107.3 |
| 2008 |  | 29.1 | 31.0 | 60.1 | 43.7 | 101.1 |  | 31.7 | 34.3 | 65.9 | 48.4 | 111.2 |  | 30.3 | 32.5 | 62.8 | 46.0 | 105.9 |
| 2007 |  | 34.2 | 24.2 | 58.3 | 69.3 | 123.6 |  | 34.4 | 35.0 | 69.4 | 51.2 | 117.1 |  | 34.3 | 29.3 | 63.7 | 60.8 | 120.0 |
| 2006 |  | 30.4 | 32.2 | 62.6 | 48.0 | 107.5 |  | 30.3 | 25.6 | 55.9 | 47.0 | 100.3 |  | 30.4 | 29.1 | 59.4 | 47.5 | 104.1 |
| 2005 |  | 31.7 | 31.9 | 63.6 | 48.8 | 109.3 |  | 29.0 | 35.7 | 64.7 | 59.0 | 119.0 |  | 30.4 | 33.8 | 64.3 | 53.7 | 114.5 |
| 2004 |  | 36.2 | 28.2 | 64.4 | 53.6 | 114.5 |  | 32.5 | 44.4 | 77.0 | 52.5 | 125.0 |  | 34.5 | 35.7 | 70.2 | 53.1 | 119.6 |
| 2003 |  | 43.8 | 39.6 | 83.4 | 59.4 | 137.9 |  | 37.1 | 32.8 | 70.0 | 64.0 | 129.5 |  | 40.6 | 36.4 | 77.0 | 61.3 | 133.6 |
| 2002 |  | 34.2 | 42.6 | 76.9 | 60.6 | 132.8 |  | 37.0 | 41.3 | 78.3 | 75.1 | 147.5 |  | 35.6 | 42.0 | 77.5 | 67.6 | 139.8 |
| 2001 |  | 49.5 | 52.1 | 101.6 | 76.9 | 170.7 |  | 32.6 | 45.7 | 78.3 | 83.7 | 155.0 |  | 41.3 | 49.2 | 90.5 | 79.7 | 163.0 |
| 2000 |  | 50.7 | 43.2 | 93.9 | 91.4 | 176.7 |  | 42.1 | 56.6 | 98.7 | 87.5 | 177.5 |  | 46.8 | 49.4 | 96.2 | 89.5 | 177.1 |

**B.4. Conclusions about the gold-standard survey**

The analysis of the gold-standard survey suggests that the survey is of good quality to support validation of the RRT methods. Analyses indicate levels and trends of under-five mortality rate that are similar in the two RRT districts. Under-five mortality rate was estimated at 102 in Balaka and 110 in Salima in 2010. The rate appeared to have decline rapidly between 2000 and 2006 and stalled since 2006 in both districts. Neonatal mortality was lower in Balaka (34 per 1000) compared to Salima (44 per 1000) in 2010. It also appeared to have decline between 2000 and 2006 but seemed to be increasing since 2006 in both district.
