# Supplementary material for: Monitoring Child Mortality through Community Health Worker Reporting of Births and Deaths in Malawi: Validation against a Household Mortality Survey
Source: PLoS One. 2014 Feb 18;9(2):e88939. doi: 10.1371/journal.pone.0088939 (PMC3928330; doi:10.1371/journal.pone.0088939)
Supplement: Web Annex S3 — Monthly number of births and neonatal, infant and under-five deaths reported by HSAs. (DOCX) [file pone.0088939.s004.docx]

**Web Annex 3**

**Monthly number of births and neonatal, infant and under-five deaths reported by HSAs**

| **Year and month** | **Number of births** | |  | **Number of neonatal deaths** | |  | **Number of infant deaths** | |  | **Number of under-five deaths** | |  |
| --- | --- | --- | --- | --- | --- | --- | --- | --- | --- | --- | --- | --- |
|  | **Balaka** | **Salima** |  | **Balaka** | **Salima** |  | **Balaka** | **Salima** |  | **Balaka** | **Salima** |  |
| **2010** |  |  |  |  |  |  |  |  |  |  |  |  |
| January | 112 | 182 |  | 0 | 1 |  | 1 | 5 |  | 7 | 11 |  |
| February | 117 | 163 |  | 2 | 1 |  | 4 | 6 |  | 7 | 10 |  |
| March | 140 | 161 |  | 3 | 3 |  | 8 | 10 |  | 20 | 15 |  |
| April | 145 | 169 |  | 10 | 5 |  | 12 | 9 |  | 24 | 16 |  |
| May | 151 | 182 |  | 2 | 1 |  | 5 | 5 |  | 11 | 11 |  |
| June | 172 | 205 |  | 1 | 7 |  | 3 | 18 |  | 13 | 24 |  |
| July | 163 | 227 |  | 7 | 5 |  | 9 | 14 |  | 17 | 31 |  |
| August | 213 | 231 |  | 7 | 19 |  | 12 | 20 |  | 15 | 36 |  |
| September | 190 | 220 |  | 8 | 7 |  | 9 | 15 |  | 15 | 20 |  |
| October | 198 | 202 |  | 3 | 5 |  | 7 | 11 |  | 13 | 14 |  |
| November | 174 | 193 |  | 3 | 3 |  | 8 | 9 |  | 10 | 13 |  |
| December | 151 | 203 |  | 2 | 3 |  | 3 | 6 |  | 10 | 8 |  |
| **2011** |  |  |  |  |  |  |  |  |  |  |  |  |
| January | 161 | 198 |  | 8 | 2 |  | 11 | 5 |  | 18 | 12 |  |
| February | 130 | 173 |  | 2 | 2 |  | 4 | 5 |  | 13 | 9 |  |
| March | 137 | 195 |  | 2 | 3 |  | 5 | 5 |  | 9 | 6 |  |
| April | 147 | 176 |  | 2 | 3 |  | 3 | 4 |  | 5 | 5 |  |
| May | 108 | 183 |  | 1 | 1 |  | 1 | 3 |  | 2 | 8 |  |
| June | 141 | 154 |  | 3 | 1 |  | 5 | 1 |  | 8 | 5 |  |
| July | 131 | 224 |  | 1 | 5 |  | 3 | 7 |  | 3 | 8 |  |
| August | 136 | 206 |  | 4 | 3 |  | 4 | 5 |  | 5 | 6 |  |
| September | 137 | 206 |  | 1 | 3 |  | 4 | 4 |  | 4 | 6 |  |
| October | 116 | 144 |  | 6 | 2 |  | 6 | 6 |  | 7 | 7 |  |
| November | 111 | 184 |  | 4 | 5 |  | 5 | 9 |  | 6 | 11 |  |
| December | 118 | 188 |  | 2 | 0 |  | 4 | 4 |  | 6 | 7 |  |
